# Citrus Genetic Engineering for Disease Resistance: Past, Present and Future

**DOI:** 10.3390/ijms20215256

**Published:** 2019-10-23

**Authors:** Lifang Sun, Nasrullah, Fuzhi Ke, Zhenpeng Nie, Ping Wang, Jianguo Xu

**Affiliations:** 1 Institute of Citrus Research, Zhejiang Academy of Agricultural Sciences, Taizhou 318026, China; 2 National Center for Citrus Variety Improvement, Zhejiang Branch, Taizhou 318026, China; 3 Department of Plant Biology and Ecology, College of Life Sciences, Nankai University, Tianjin 300071, China

**Keywords:** genetic engineering, citrus, disease resistance, breeding

## Abstract

Worldwide, citrus is one of the most important fruit crops and is grown in more than 130 countries, predominantly in tropical and subtropical areas. The healthy progress of the citrus industry has been seriously affected by biotic and abiotic stresses. Several diseases, such as canker and huanglongbing, etc., rigorously affect citrus plant growth, fruit quality, and yield. Genetic engineering technologies, such as genetic transformation and genome editing, represent successful and attractive approaches for developing disease-resistant crops. These genetic engineering technologies have been widely used to develop citrus disease-resistant varieties against canker, huanglongbing, and many other fungal and viral diseases. Recently, clustered regularly interspaced short palindromic repeats (CRISPR)-based systems have made genome editing an indispensable genetic manipulation tool that has been applied to many crops, including citrus. The improved CRISPR systems, such as CRISPR/CRISPR-associated protein (Cas)9 and CRISPR/Cpf1 systems, can provide a promising new corridor for generating citrus varieties that are resistant to different pathogens. The advances in biotechnological tools and the complete genome sequence of several citrus species will undoubtedly improve the breeding for citrus disease resistance with a much greater degree of precision. Here, we attempt to summarize the recent successful progress that has been achieved in the effective application of genetic engineering and genome editing technologies to obtain citrus disease-resistant (bacterial, fungal, and virus) crops. Furthermore, we also discuss the opportunities and challenges of genetic engineering and genome editing technologies for citrus disease resistance.

## 1. Introduction

The genus *Citrus* and related genera (*Fortunella*, *Poncirus*, *Eremocitrus,* and *Microcitrus*), representing one of the most widely grown fruits, belong to the angiosperm subfamily Aurantioideae of the Rutaceae family [1]. The fruits of citrus are rich in many nutrients, especially vitamin C, and hence constitute important parts of the daily diet and world fruit crops. Disease, drought, cold, and soil salinity are the main factors that can limit citrus production, among which, disease is especially significant, such as citrus canker, huanglongbing (HLB, citrus greening), and other fungal or viral diseases [2,3]. Diseases (bacterial, fungal, or viral) can appear in a region, and within a few years, can spread and have a major economic impact. Various approaches, such as the use of chemical pesticides and other synthetic molecules, have been used to control diseases in crop plants, including citrus [4,5]. However, the side effects of these chemicals should not be ignored, such as an increased pesticide resistance in the pathogens, resurgence, pesticide residues in agricultural products, environmental pollution, and ecological balance issues [6]. Therefore, durable disease resistance is an important aim in each breeding program, and developing disease-resistant cultivars is a prime objective of breeders. Traditional breeding methods have been successfully used to improve citrus cultivars and develop new varieties in the past, but this has been done with difficulty and limitations due to the large plant size and long juvenility of this crop, incompatibility, polyembryony, heterozygosity, and parthenocarpy, etc. [7,8]. Hence, in traditional breeding, it is difficult to improve the desired traits of citrus in the short term. Additionally, traditional breeding is mainly restricted to the traits related to fruit quality, such as the fruit ripening time, flesh color, and seed number [9].

Genetic engineering is the deliberate modification of the characteristics of an organism by the manipulation of its genetic material, which has been described as a new technological paradigm [10]. The main genetic engineering technology is based on transgenesis that followed the discovery of the recombinant DNA technique, which allows plant breeders to cross crop species and introduce genes from non-related plants and other organisms into crop plants [11,12]. The fundamental strategy in genetic engineering is to modify plants so that they are productive in adverse conditions caused by biotic and abiotic stress, for instance, pathogens, pests, drought, saline, and unfertile environments [8]. Generally, genetic engineering via transformation is an alternative method to incorporate desirable traits in plants in a short cycle, with a high efficiency and easy control. Since the first reports of transgenic plants appeared in 1984 [13], there has been very rapid progress directed at using this new technology for the practical end of crop improvement. In recent years, genetic manipulation has been employed as a new route to overcome the intrinsic barriers of traditional techniques, and genetic engineering methods based on the introduction of transgenes and development of transgenic plants have been successfully adopted to improve crops [14]. Transgenic crops have gained attention worldwide since they have emerged. By introducing bioengineering technology in crop-breeding issues, genetically modified crops with an improved quality, enhanced resistance to biotic or abiotic stresses, increased yield, or reduced harmful components have been generated [15,16,17].

During the last two or three decades, genetic engineering methods based on the use of transgenes have been successfully adopted to improve fruit plants and have been mainly focused on an enhanced tolerance to biotic and abiotic stresses, increased fruit yield, improved post-harvest shelf life, reduced generation time, and production of fruit with a higher nutritional value [18]. Many successful transformation events have been achieved and different genes have been introduced into citrus that comprise antibiotic and reporter genes, genes that shorten the juvenile phase, genes that confer stress tolerance and disease resistance, and fruit quality-related genes [19,20,21,22,23].

Among all the techniques, genetic engineering has been successfully used to obtain enhanced disease-resistant citrus, which suggests that genetic engineering is an efficient approach for the development of disease-resistant citrus cultivars. In this review article, we attempt to summarize the recent successful progress that has been achieved in the effective application of genetic engineering and genome-editing technologies to obtain citrus disease-resistant (bacterial, fungal, and virus) crops. Furthermore, the opportunities and challenges of genetic engineering and genome editing technology for citrus disease resistance are also discussed here.

## 2. Genetic Engineering History of Citrus Crops

**Figure 1:**
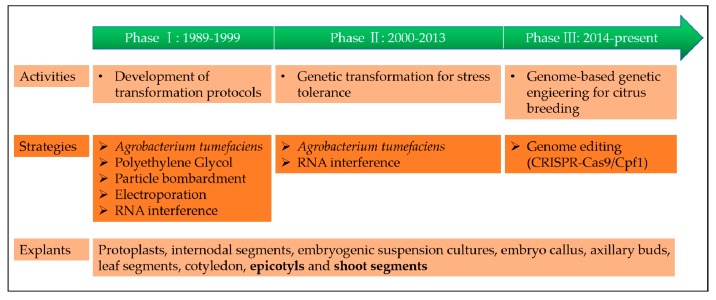
History of the genetic transformation of citrus.

Tremendous progress has been made in both our scientific understanding and technological capabilities since the first report of a genetically engineered crop conferring resistance to disease, which was a virus-resistant tobacco expressing a viral coat protein gene [24]. For a better understanding of citrus genetic engineering history, we have divided it into three phases, based on major advances (Figure 1).

### 2.1. Phase I: Development of Transformation Protocols (1989–1999)

After the development of the polymerase chain reaction (PCR) in 1983 by Kary Mullis, a revolutionary method, the development of genetic transformation protocols, has been an attractive field of research. For citrus genetic transformation, several protocols have been developed. Kobayashi and Uchimiya made the first attempt to use a citrus protoplast to develop genetically modified plants; however, Vardi et al. reported the first successful attempt by generating a transgenic citrus plant through a polyethylene glycol (PEG) mediated direct DNA transfer method [25,26]. Subsequently, *Agrobacterium*-mediated transformation was developed by Hidaka et al. and has become the most widely used gene transfer method in citrus since [27]. Besides the indirect gene-transfer methods, some successful studies have been performed by direct gene-transfer methods using electroporation or particle bombardment [28]. Yao et al. reported the first successful transformation of citrus embryogenic cells using particle bombardment, which was reported in tangelo (*C. reticulata* × *C. paradisi*) [29]. To optimize the efficiency of these transformation protocols/methods, many studies have continually been published for different citrus species.

### 2.2. Phase II: Genetic Transformation for Stress Tolerance (2000–2013)

To develop stress-tolerant citrus for both biotic and abiotic stresses, *Agrobacterium*-mediated transformation has been the most widely used gene transfer method in citrus. In order to obtain a high degree of genetic transformation precision, different types of citrus explants have been used, such as internodal segments, embryogenic suspension cultures, embryo callus, axillary buds, leaf segments, cotyledon, epicotyls, and shoot segments [9,30,31,32,33,34,35,36,37]. Most of the achievements made through *Agrobacterium*-mediated transformation have been cited in later parts of the paper. Recently, RNA interference (RNAi) protocols have been used to develop transgenic resistance to citrus tristeza virus (CTV) by silencing the expression of critical genes in CTV-infected cells [38,39,40]. 

### 2.3. Phase III: Clustered Regularly Interspaced Short Palindromic Repeats (CRISPR)/CAS (CRISPR-Associated) Systems (2014–Present)

Genome editing technology, a way to make precise changes to the genomic DNA of a cell or organism, have become a powerful tool for the precise manipulation of targeted genome sequences in crops [41,42,43]. Currently, there are three major types of sequence-specific nucleases for genome editing: zinc finger nucleases (ZFNs) and transcription activators such as effector nucleases (TALENs) and the clustered regularly interspaced short palindromic repeats (CRISPR)/Cas (CRISPR-associated protein) system [43]. In comparison to ZFNs and TALENs, the CRISPR/Cas9 system is widely used in laboratories because of its simplicity, design flexibility, and high efficiency. Recently, the CRISPR/Cas system has been successfully reported for citrus, with some good results for enhancing disease resistance [44,45,46,47,48,49]. More details will be discussed later in the paper in Section 5.

## 3. Advances in Transgenic Research for Bacterial Disease Resistance in Citrus

Genetic transformation is one of the important methods of choice for protecting susceptible citrus cultivars against canker or HLB caused by related bacterial pathogens in a shorter time. Therefore, transgenic approaches introducing exogenous genes, such as plant resistance genes, key positive regulators of SAR genes, antimicrobial peptide genes, plant metabolic genes, pathogenic genes, and kinase genes, have been applied to generate transgenic citrus crops resistant to canker or HLB infections through *Agrobacterium*-mediated transformation. All the successful transformation events for improving bacterial disease resistance in citrus are discussed and listed below.

### 3.1. Transgenic Research Related to Canker Resistance

Citrus canker, caused by the bacterial pathogen *Xanthomonas citri* ssp. *citri* (*Xcc*), is one of the most destructive citrus cultivar diseases reported all over the world [50]. Citrus canker affects citrus production, leading to yield losses, a poor fruit quality, and trade barriers [51]. Strategies like eradication and pathogen exclusion have been mainly used to manage the disease [5]. At present, cultural practices and chemical controls are the main methods used to manage citrus canker. Among the chemicals, copper-based chemicals have been expressed an adequate control of *Xcc* due to prolonged residual activity compared to other contact bactericides [52,53], such as copper oxychloride, copper hydroxide, copper sulphate, and ammonia-copper carbonate, which have been found to be highly effective against *Xcc* [54]. However, a continuous reliance on these compounds can cause mutations and the emergence of aggressive races of *Xcc* [52,53]. In addition, these methods are expensive and harmful to the environment. Hence, the use of resistant cultivars would be a better method to control citrus canker and resistance might be introduced through cross breeding. However, there have been no reports about the development of resistant citrus cultivars via conventional breeding. Genetic engineering may be a better method of improving disease resistance to citrus canker [55].

To improve the resistance to canker in citrus by transgenic approaches, different strategies have been proposed, including the over-expression of genes that code for antibacterial peptides, disease-resistance proteins, the kinase gene, transcription factors, and other exogenous genes of a plant/non-plant origin that enhance natural plant defenses (Table 1). Antimicrobial peptides are important components of innate immune defense against microbial pathogens in a wide range of organisms [56,57]. To obtain resistant cultivars, different antimicrobial peptide genes, such as *attacin* A, *Shiva* A, *Cecropin* B, *Stx IA*, *D2A21*, and the dermaseptin gene, have been introduced into citrus cultivars and rootstock to enhance canker resistance [35,58,59,60,61,62,63], the results of which indicate that the over-expression of these exogenous peptide genes significantly enhances their canker disease resistance. The *Arabidopsis NPR1* gene (*AtNPR1*) has been well-established as a key positive regulator of systemic acquired resistance (SAR), which acts downstream of the signal molecule SA. Over-expression of the *AtNPR1* gene or its orthologs also enhances disease resistance in many crop plants including rice, wheat, rapeseed, tomato, and apple [64,65,66,67,68], which makes AtNPR1 a workable target for the genetic engineering of non-specific resistance in plants. This kind of broad-spectrum disease resistance gene was also introduced into citrus to enhance canker resistance. Two previous reports showed that transgenic grapefruit and sweet orange that over-express the positive regulator of SAR, the *AtNPR1* gene, or its homologous gene *CtNH1* from *Citrus maxima*, were less susceptible to *Xcc* [55,69]. The introduction of resistance genes (R-genes) is one of the strategies used to improve the plant’s resistance to pathogens [70]. Disease-resistant R-genes are frequently used in breeding for crop protection. The *Bs2* gene is a member of the nucleotide binding site-leucine-rich repeat (NBS-LRR) class of R genes, which has been shown to confer resistance against pathogenic strains of *Xanthomonas campestris* pv. *vesicatoria* (*Xcv*) in susceptible pepper, tomato, and tobacco plants [71]. It has been reported that the over-expression of *Bs2* from pepper leads to a decreased susceptibility to *Xcc* [72,73]. Another R gene, *Xa21*, first cloned from the wild rice *Oryza longistaminata*, encodes a receptor kinase-like protein that consists of LRR [74]. Likewise, transgenic citrus with the rice *Xa21* gene showed less susceptibility to *Xcc* [75,76,77,78]. Reactive oxygen species (ROSs) have emerged as important regulators of plant stress responses, and were observed in a wide range of plant–pathogen interactions involving bacteria, fungi, and viruses [79]. The accumulation of ROSs was proposed as the earliest event induced during plant–pathogen interaction, which controls and inhibits pathogen growth. Transient elevations in ROS levels can enhance stress tolerance by activating the defense mechanisms, including kinases and components of the signaling network [80]. Over-expression of the pathogen-associated molecular pattern (PAMP) receptor *NbFLS2* can increase ROS production and activate PAMP-triggered immunity and defense-associated gene expression in citrus, the results of which showed that the integration and expression of the *NbFLS2* gene can increase canker resistance [81]; the mitogen-activated protein kinase gene *CsMAPK1* functions in the citrus canker defense response through the activation of defense-related gene expression and ROS production during infection, leading to a reduction in canker symptoms and a decrease in bacterial growth [82].

Other exogenous genes, such as hairpin gene *hrp*N, spermidine synthase gene *MdSPDS1*, transcription factor *terf1*, pathogenesis gene *pthA-nls*, and cysteine-rich peptide *theonin*, have also been introduced into citrus, and all the results showed that the transgenic lines of citrus with these genes were less susceptible to canker disease [83,84,85,86,87]. Therefore, the over-expression of exogenous genes in citrus is a promising approach for the development of cultivars that are more resistant to citrus canker.

### 3.2. Transgenic Research for Huanglongbing Resistance

Huanglongbing (HLB), another devastating citrus disease worldwide, is also known as citrus greening. HLB is mainly associated with phloem-limited bacteria that belong to the *Candidatus Liberibacter* genus, including *Candidatus Liberibacter asiaticus* (CaLas), *Candidatus Liberibacter americanus* (CaLam), and *Candidatus Liberibacter africanus* (CaLaf) [88,89,90]. The insect vector responsible for the transmission of these phloem bacteria can be either the Asian citrus psyllid, *Diaphorina citri*, or the African citrus psyllid, *Trioza erytreae* [91]. HLB infection often leads to a drastic reduction in the quantity and quality of citrus fruits and eventually renders the infected trees useless [91,92]. The major strategies for managing and controlling HLB disease have been summarized in previous literature, including the vector control of psyllid populations (chemical and biological control), use of antimicrobials, thermotherapy, use of disease-free planting materials, and nutrient enhancement of the trees [90,93]. Among these options, chemical control to reduce psyllid and *Ca. Liberibacter* populations has been used broadly. From the diverse set of compounds tested, ampicillin, carbenicillin, penicillin, cefalexin, oxytetracycline, streptomycin sulfate, rifampicin, and sulfadimethoxine have been shown to be highly effective in suppressing HLB [94]. However, the usage of antibiotics needs to continue over time to control HLB over the long term, which also introduces problems associated with operation costs and potentially adverse environmental effects. Furthermore, almost all commercial citrus cultivars are susceptible to HLB. Therefore, the best solution for the management of HLB is to develop resistant or tolerant cultivars of important citrus species through genetic engineering, which remains the fastest method for the improvement of existing citrus cultivars [95].

In a previous report, it was found that transgenic citrus lines expressing the broad-spectrum disease-resistance gene *AtNPR1* in citrus increase resistance to citrus canker [55]. Recent results from two reports show that over-expressing *AtNPR1* can also provide resistance to HLB. Dutt et al. showed that some transgenic Hamlin and Valencia sweet orange lines that over-express the *AtNPR1* gene, under a constitutive *CaMV35S* promoter and a phloem-specific *AtSUC2* promoter, exhibited reduced disease severity and a few lines remained disease-free, even after 36 months of planting in a high-disease pressure field site [22]. Results from Robertson et al. also indicate that the expression of high levels of *AtNPR1* in citrus can provide tolerance to HLB under strong disease pressure in the greenhouse [89]. These results are also in agreement with the findings reported by Wang et al. [96], who showed that four *AtNPR1-*like genes were expressed to significantly higher levels in the HLB-tolerant genotype than in the HLB-susceptible genotype, by evaluating transcriptome differences between two closely related cultivars (HLB-tolerant Jackson grapefruit-like hybrid and HLB-susceptible Marsh grapefruit trees) after HLB infection. All these studies indicate that *AtNPR1-*like gene-mediated defense signaling may contribute to the HLB-tolerant phenotype [96]. In addition, three other exogenous-resistant genes were separately introduced into the citrus, all of which improved the resistance to HLB. The expression of antimicrobial gene *attacin* A (*attA*) in sweet orange Hamlin and Pêra showed significantly fewer symptoms of HLB compared to non-transgenic plants of this cultivar [97,98]. A modified thion was introduced into citrus and the transgenic citrus showed a stronger resistance to HLB and citrus canker [87]. *Cecropin* B, an antimicrobial peptide from Chinese oak silk worm, was expressed under the phloem-specific promoter *GRP1.8* from the French bean to reduce the susceptibility of Tarocco blood orange to HLB [99].

## 4. Advances in Transgenic Research for Fungal and Viral Disease Resistance in Citrus

New approaches and research in genetic engineering have provided novel opportunities for the generation of plants for resistance against funguses and viruses outside of conventional breeding methods. In addition to canker and HLB diseases, attention is now also being given to other fungal and viral diseases in citrus, such as citrus tristeza virus (CTV), citrus psorosis (CP), root rot and gummosis, mal secco, gray mold, black spot, and citrus scab. Resistance to these fungal and viral diseases has also been enhanced by transgenic technology (Table 2).

CTV, one of the most important viral diseases affecting citrus, causes a quick decline of most citrus species, as well as a reduction in the fruit yield and quality of some cultivars [100,101]. Due to the agronomic characteristics of citrus cultivars, genetic transformation appears to be the most promising technique for developing CTV resistance, and the possibility of creating transgenic plants with an enhanced resistance to CTV has been evaluated using different gene constructs and citrus genotypes [101]. The pathogen-derived resistance (PDR) strategy has been successfully used in developing CTV tolerance or resistance materials by introducing genes or selected segments of the CTV genome in citrus plants. Transgenic Mexican limes that express *p25* and *p23* were obtained by genetic engineering and found to show a significant delay in virus accumulation and no CTV symptoms, so were capable of conferring resistance to CTV [102,103]. Ananthakrishnan et al. found that grapefruit expressing *p23* and 3′-UTR exhibited reduced CTV replication in protoplasts [104]. Muniz et al. transformed two sweet oranges with *p25*, intron-spliced hairpin *p25* and a 559-nt-long 3′-terminal conserved region, respectively, and found that some lines might have partial repressing effects on virus replication [105]. In some cases, expression of the CTV coat-protein gene led to an increase in CTV resistance. Protoplasts isolated from ten sweet orange callus lines genetically transformed with the CTV-392/393 sequence from the CTV genome exhibited different CTV replication levels [106]. Additionally, Cervera et al. reported the first transgenic Mexican lime by the ectopic expression of single-chain variable fragment (scFv) recombinant antibodies, and most transgenic lines displayed resistance or tolerance after being challenged by CTV inoculation [107]. RNA silencing or RNA interference (RNAi) has been found to be the most important mechanism that plants use to combat viral infections [108]. The main strategy of RNAi for obtaining viral resistance is to express viral sequences that will form self-complementary hairpin RNA (hpRNA) in the host when expressed [109]. In citrus, nine lines of transformed Mexican lime with sense, antisense, and intro-hairpin versions of the 549-nt-long 3′-terminal of CTV were found to show CTV resistance [38]. It was reported that three transgenic Mexican lime lines expressing an untranslatable version of the three CTV-silencing suppressor genes in the intron-hairpin version showed complete resistance to CTV-T36 infection under laboratory conditions [39]. Similarly, some lines of transformed sour orange with a hairpin-structured *p20* conservative region showed resistance or tolerance to severe CTV strains [109,110].

Similar work has also been done to enhance the resistance of other citrus pathogens by researchers. Two transgenic sweet orange lines containing different genes of citrus psorosis virus (CPsV) were obtained separately, both of which showed a delay in CPsV symptoms [111,112]. Furthermore, intron-hairpin RNA transcripts corresponding to CP genes (*ihpCP*) of CPsV were introduced into sweet orange, leading to the regeneration of transgenic plants expressing ihpRNA with enhanced CPsV resistance [113]. Various endochitinase genes, such as *chit42* from *Trichoderma harzianum*, have also been successfully transformed and expressed to impart an increased fungal tolerance in lemon. Transgenic lemon with *chit42* showed significantly less lesion development after inoculation with *Phoma tracheiphila* and *Botrytis cinerea*, the causal agents of mal secco and gray mold in citrus [114,115]. A gene for the PR-5 protein from tomato has been expressed in transgenic sweet orange and regenerants, and all the transgenic lines exhibited significant protection against *Phytophthora citrophthora*, which causes root rot and gummosis [116]. Several transgenic Duncan grapefruit lines expressing the antimicrobial *Attacin E* gene had a significantly lower susceptibility to *Elsinoë fawcettii* compared to the non-transformed control, and significant activity against citrus scab was unexpectedly found [117]. Additionally, regulation of the level of D-limonene in citrus fruit by a transgenic approach was attempted to enhance the resistance against pathogens such as black spot [118].

## 5. Application of Genome-Editing Techniques in Disease-Resistance Breeding of Citrus

Genome editing, a specific and efficient tool for generating useful novel phenotypes, surely represents a great technical innovation in plant breeding. Generally, genome-editing technology employs three types of engineered endonucleases: zinc finger nucleases (ZFNs), transcription activator-like effector nucleases (TALENs), and clustered regularly interspaced short palindromic repeats and CRISPR-associated proteins (CRISPR/Cas) for site-specific cleavage and the emerging CRISPR/Cas9 is comparatively easy to prepare, affordable, and can be scaled up better than ZFNs and TALENs [119,120,121]. Though developed recently, CRISPR/Cas9 technology has already been established in several important plant species through gene mutation, repression, activation, and epigenome editing, such as rice [122], wheat [123], maize [124], and some horticultural crops, including tomato, petunia, citrus, grape, potato, carrot, and apple [125,126,127,128,129]. Using this technology, many agronomically important traits, such as heat/cold tolerance, disease resistance, herbicide tolerance, and yield improvement, have been introduced in plants [119,130]. CRISPR/Cas9 technology has been efficiently applied in developing disease resistance to many viruses [131,132]. In a previous report, CRISPR/Cas9 was successfully used to incorporate the mutagenesis insusceptible genes *MLO-7* in grape cultivar Chardonnay and *DIPM-1*, *DIPM-2*, and *DIPM-4* in apple cultivar Golden delicious, to increase their resistance to powdery mildew and fire blight disease, respectively [133].

In citrus, the CRISPR/Cas9 system was firstly used to target the *CsPDS* gene in sweet orange and Duncan grapefruit and successfully modified the *CsPDS* gene via *Xcc*-facilitated agro infiltration, an optimized transient expression method [44,134]. As expected, the modified *CsPDS* sequence expressing Cas9/sgRNA was not detected in sweet orange leaves [44], which indicated that CRISPR/Cas9 successfully induced mutations in the targeted gene in citrus. *CsLOB1*, a member of the lateral organ boundaries domain (LBD) family of transcription factors, was previously identified as a critical citrus disease susceptibility gene for citrus canker [135]. The strain of *Xcc*, bacteria that causes citrus canker, encodes transcription activator-like (TAL) effector *PthA4* that binds to the effector-binding elements (EBE) in the promoter of *CsLOB1* and activates the expression of this canker-susceptibility gene [135]. Interestingly, CRISPR/Cas9-mediated modification of the EBE of one single allele of the *CsLOB1* gene in Duncan grapefruit alleviated the canker symptoms slightly [45]. However, mutation of the EBEs of both alleles of *CsLOB1* promoters showed a high degree of resistance to citrus canker in Wanjincheng orange [47]. Furthermore, another study showed that editing the coding region of *CsLOB1* via CRISPR/Cas9 also provided resistance to canker in Duncan grapefruit [46]. In a recent report, *CsWRKY22*, a marker gene for pathogen-triggered immunity in Wanjincheng orange, was knocked out by the CRISPR/Cas9 system, and the mutant plants showed a significantly decreased susceptibility to citrus canker [49]. In addition, CRISPR/Cas12a (Cpf1), another improved CRISPR/Cas system, has been employed to edit the Duncan grapefruit gene *CsPDS* with a higher efficiency and lower off-target effects, which will enhance the scope of citrus genome editing [48]. These studies indicate that CRISPR-mediated genome editing can be a promising pathway to generate disease-resistant citrus cultivars. Due to the vague virulence mechanisms of HLB and other diseases, the CRISPR/Cas system cannot achieve the goal of conferring resistance to citrus HLB and other diseases at present. However, researchers are now attempting to use the genome editing system to create citrus cultivars that are less susceptible to HLB [136,137].

Besides the recent successes, the CRISPR/Cas system faces several limitations and challenges in citrus. For instance, the most debated challenges in the CRISPR/Cas system are its potential off-target mutations and unintended on-target changes. In citrus, its polyploidy nature poses a challenge for the CRISPR/Cas system, because it has been proved that the gene-editing efficiency is usually lower in polyploidy plants than diploids, as multiple alleles must be edited simultaneously [45,47,138]. Therefore, further research is needed to increase the efficiency of tissue culture-based *Agrobacterium*-mediated delivery of the CRISPR/Cas system efficiency in different citrus species. However, in a recent report, Hongkong kumquat (*Fortunella hindsii*), a wild citrus species with a short juvenility, monoembryony, and applicability of CRISPR, is being exploited as a model species for citrus research [139].

## 6. Genetic Approaches to Suppress Vector-Borne Bacterial/Viral Diseases in Citrus

Recently, several reports have shown the great potential of RNAi and CRISPR to develop novel management strategies of vector-borne viral/bacterial diseases in citrus [140,141]. For instance, HLB diseases have been successfully controlled by applying RNAi-mediated protection against the *Candidatus* Liberibacter asiaticus (CLas) bacteria vector African citrus psyllid (ACP) *Diaphorina citri* through bioassays using plant feeding systems [142,143,144,145,146,147,148], detached leaf bioassays [149,150], leaf disc, artificial diet, sugar solutions [151]; topical applications and soaking [152,153,154]. All the results showed that the ACP was very sensitive to ingested dsRNA, causing significant suppression of the targeted transcript and increased psyllid mortality. Additionally, the application of RNA suppression using antisense oligonucleotides, such as 2’F-ANA-ASO/ASO and cell-penetrating peptide morpholinos (PPMOs), was also reported to trigger RNAi degradation for suppressing pests and pathogens in infected citrus seedling trees, which significantly reduced ACP and CLas bacteria [140]. CRISPR-Cas9 was used to conduct a knockout of the thioredoxin gene in ACP, and the CRISPR-Cas9-treated psyllids resulted in psyllids with longer development times, shorter adult lifespans, and a reduced fecundity [140,155]. Interestingly, CRISPR/Cas system was ever found in the CLas genera [156], which could be used to develop effective strategies for controlling the HLB pathogen.

Besides HLB, CTV and citrus variegated chlorosis (CVC) diseases have also been reduced by RNAi-mediated protection strategies against *Aphis* (*Toxoptera*) *citricidus* (Kirkaldy) [157,158], and *Homalodisca vitripennis* [159,160] in citrus. For citrus canker, recently, two studies have shown that the action mode of the main TAL effector PthA4/PthA4^AT^ from *Xcc* may provide new clues for controlling canker by interfering with the bacteria *Xcc* [161,162]. Therefore, RNAi and CRISPR could definitely be exploited to develop novel management strategies to suppress the vectors and pathogens, thereby bringing benefits to both growers and consumers without gene-editing plants.

## 7. Conclusions and Future Perspectives

Due to some of the biological characteristics of citrus, conventional breeding methods have demonstrated limitations to producing new citrus cultivars with an improved resistance to diseases. Therefore, genetic engineering, including transgenic or genome-editing technologies, can be a method of choice to overcome the limitations posed by traditional breeding and have made it possible to protect susceptible commercial cultivars against pathogens, which allows the release of improved cultivars with desirable characteristics in a shorter time. Exogenous genes, such as plant-resistance genes, key positive SAR regulator genes, insect antimicrobial peptide genes, plant metabolic genes, pathogenic genes, and kinase genes, have been introduced in citrus through *Agrobacterium*-mediated genetic transformation, and transgenic lines with an excellent resistance to canker have been obtained (Table 1). Furthermore, the broad-spectrum disease-resistance gene *AtNPR1*, a modified plant thionin gene, and antimicrobial peptide genes *Cecropin* B and *attacin* A, have been respectively introduced into citrus, and all effectively improved the resistance to HLB. These genes of a plant or non-plant origin encoding antibacterial proteins, such as plant resistance genes (R-genes), regulators of SAR genes, antimicrobial peptide genes, etc., have been introduced in citrus to inhibit bacterial pathogenicity factors though various genetic techniques. Transgenic plants that constitutively express proteins with potential antibacterial or antifungal activity can reduce the activities of specific soil-borne plant pathogens in the rhizosphere, and affect specific plant-beneficial components of the rhizosphere microflora [163,164], resulting in changes of citrus disease resistance. Antimicrobial peptides (AMPs), especially, are important components of the innate immune defense system against microbial pathogens [165], for which the modes of action in transgenic plants may involve interactions between the peptides and bacterial membrane, leading to bacterial membrane disruption, cytoplasmic leakage, and interference with intracellular macromolecule synthesis to rapidly kill the bacteria [165,166]. Besides the main bacterial diseases, resistance to other fungal and viral diseases in citrus, including anthracnose, gray mold, scab, black spot, root rot and gummosis, tristeza virus, and psorosis, were also enhanced by transgenic technology (Table 2). However, conventional biomolecule delivery methods in plants, including electroporation, biolistics, *Agrobacterium*-mediated delivery, and cationic delivery, have critical drawbacks, such as a low efficiency, narrow species range, limited cargo types, and tissue damage [167]. Therefore, improving the existing delivery systems and developing new systems will be key to reducing barriers to the inexpensive application of genetic engineering in plants, especially genome editing. Nanomaterials have unique and tunable physical and chemical properties, which can interact with biological matter with exquisite control and precision [168]. Nanoparticles can be used as a carrier system to deliver the genetic materials, such as plasmid DNA, RNA, and oligonucleotides into cells efficiently and rapidly, which reduce the drawbacks and limitations associated with current *Agrobacterium*-mediated transgene delivery systems [167,168,169]. A few successful examples show promise for nanoparticle-mediated passive delivery to plants in vitro [170,171,172] and in vivo [173,174], indicating the potential for passive nanoparticle-mediated delivery with a high efficiency and low toxicity. In the future, nanotechnology-mediated delivery system can overcome the delivery challenges and enhance the efficacy of citrus genetic engineering.

Novel genome-editing systems help introduce stably inherited point modifications in the plant genome and allow the creation of non-transgenic plants [175,176], which may meet the challenges faced by citrus breeders, such as the quest to develop productive, disease-resistant varieties with tasty, high-quality, and nutrient-packed fruit. In contrast to ZFNs and TALENs, second-generation genome editing techniques like CRISPR-Cas9 and CRISPR/Cpf1 involve easier design and execution methodologies that are also more time- and cost-effective [120], both of which are currently the best studied and most widely used CRISPR systems in plants. In citrus, the canker susceptibility gene *CsLOB1* and canker immunity response gene *CsWRKY22* have been modified at the promoter region or coding region by CRISPR/Cas9 [45,46,47,49] and the citrus *CsPDS* gene was also effectively modified via a new improved CRISPR/Cpf1 system [48]. The genomes of an increasing number of citrus and disease-related bacterial species have already been sequenced or are in the process of being sequenced, which will solve the problem of a lack of genomic information and thus permit the target gene/site (susceptibility gene) to be modified using genome-editing technology. Furthermore, continuously improved genome-editing technologies with a nanotechnology-mediated delivery system will play a significant role in developing new disease-resistant citrus cultivars in the future.

Worldwide, the human population is projected to reach 9.8 billion in 2050, and it is estimated that the global food supply will need to increase by 70% till 2050 to meet the rapidly mounting population nutritional demands [177,178]. Various abiotic stresses, biotic stresses, and recent climate change waves worsen the task that the world face in terms of meeting the nutritional necessities. Genetic engineering, which refers to the direct alteration of an organism’s genetic material using biotechnology, could be an integrated and diversified approach against these challenges [179]. Genetically engineered crops in general increase crop productivity through a reduction in the cost of cultivation and crop loss caused by biotic and abiotic stresses, so provide more affordable food [180]. In addition to economic gains, genetically engineered crops reduce the number of insecticide applications, importantly contributing to a more sustainable environment. The products of genetic engineering are often referred to as GE or GMO (genetically modified organisms) [181]. Despite transgenic crops, such as soybeans, maize, cotton, and canola, having enjoyed wide commercial success around the world [182], their commercial utilization is still hampered by some legal, ethical, and experimental issues, which mostly limit their use to research. In contrast, CRISPR/Cas-mediated genome editing seems to be the most promising strategy to improve crop cultivars without introducing foreign genes, and the United States Department of Agriculture (USDA) has stated that CRISPR/Cas9-edited crops, which have the potential to be called non-GMO, can be cultivated and sold without regulatory monitoring [183,184]. However, confidence in applying genome-editing tools in agriculture remains limited. The biggest potential obstacles for the use of genome-editing technologies in agriculture are public acceptance and government regulation [185]. There is still no internationally accepted regulatory framework for gene-editing products, and different countries/agencies have different takes on the use of GMOs [185,186]. For instance, the European regulatory agencies emphasize how the plants were produced, and have recently ruled that gene-edited products/crops should be treated like traditional GMOs, which are under very strict regulation in the European Union [187,188]. By contrast, the USDA tends to focus on the end product, and they have determined that gene-edited crops are exempt from GMO regulations [184]. Furthermore, the USDA has outlined that genome-edited crops can be considered as products from biological mutagenesis, such as chemical and radiation mutagenesis widely used in conventional plant breeding. Overcoming regulatory obstacles requires public awareness and political willingness in order to strive for some form of consistency among countries to establish a clear position on genome editing technologies. The regulatory processes are often unpredictable and challenging. However, continued dialog among regulatory authorities, as well as positive actions taken by progress-oriented countries, will help to bring about improvements in the GMO product regulations.

## Figures and Tables

**Table 1:** The genes used in the genetic transformation of citrus to impart resistance to canker.

Genes	Sources	Type	Species	References
*Attacin A (attA)*	*Trichoplusia ni*	Antimicrobial peptide	*C. sinensis* cv. Hamlin/Natal/Pera/Valencia	[58,59]
*Cecropin* B and *Shiva* A	*Synthetic*	Antimicrobial peptide	*C. sinensis* (L.) Osbeck	[35]
*Stx IA*	*Sarcophaga peregrina*	Antimicrobial peptide	*C. sinensis* (L.) Osbeck	[60,61]
*D2A21*	*Synthetic*	Antimicrobial peptide	Carrizo citrange	[62]
*Dermaseptin gene*	*Synthetic*	Antimicrobial peptide	*C. sinensis* cv. Pineapple	[63]
*AtNPR1*	*Arabidopsis thaliana*	Key positive regulator of systemic acquired resistance (SAR)	*C. paradisi* Macf.; *C. sinensis* cv. Hamlin	[55]
*CtNH1*	*Citrus maxima*	Key positive regulator of SAR	*C. paradisi* Macf.	[69]
*Bs2*	*Capsicum annuum*	Resistance gene	*C. sinensis* cv. Hamlin/Natal/Pera/Valencia/Anliucheng; W. Murcott tangor	[72,73]
*Xa21*	*Oryza longistaminata*	Resistance gene	*C. limon* cv. Eureka Frost Nuclear; *C. sinensis* cv. Pineapple	[75,76,77,78]
*NbFLS2*	*Nicotiana benthamiana*	Leucine-rich repeat (LRR) receptor–like kinase gene	Carrizo citrange	[81]
*CsMAPK1*	*Citrus sinensis*	Mitogen-activated protein kinase gene	Troyer citrange	[82]
*hrp*N	*Erwinia amylovora*	Hairpin gene	*C. sinensis* cv. Hamlin/Valencia	[83]
*MdSPDS1*	*Malus domestica*	Spermidine synthase gene	*C. sinensis* cv. Anliucheng	[84]
*terf1*	*Solanum lycopersicum*	Transcription factor	*C. sinensis* (L.) Osbeck	[85]
*pthA-nls*	*Xanthomonas axonopdis* pv.citri	Pathogenesis gene	*C. sinensis* (L.) Osbeck	[86]
*Modified theonin*	*Synthetic*	Cysteine-rich peptide	Carrizo citrange	[87]

**Table 2:** The genes used for imparting resistance to fungal and viral diseases in citrus.

Genes	Sources	Type	Fungi and Virus Diseases	Species	References
*CTV-CP*	*Citrus Tristeza virus*	CTV coat protein gene	Tristeza virus	*C. sinensis* cv. Valencia/Hamlin	[105]
*CTV-392/393*	*Citrus Tristeza virus*	CTV-derived gene		*C. sinensis* cv. Itaborai	[106]
*3DF1scFv*	*Hybridoma 3DF1 cell*	Monoclonal antibody		*C. aurantifolia* (Christm.) Swing	[107]
*p20/23/25*	*Citrus Tristeza virus*	Silencing suppressor protein		*C. aurantifolia* (Christm.) Swing.; *C. paradisi* Macf. *C. sinensis* (L.) Osbeck	[38,39,102,103,104,109,110,116]
*CPsV-CP ihpCP*	*Citrus Psorosis virus*	Coat protein gene/siRNA	Psorosis virus	*C. sinensis* (L.) Osbeck;	[111,112,113]
*chit42*	*Trichoderma harzianum*	Endochitinase	Mal secco and gray mold	*C. limon* (L.) Burm. f.	[114,115]
*PR-5*	*Solanum lycopersicum*	Pathogenesis-related protein	Root rot and gummosis	*C. sinensis* cv. Pineapple	[116]
*Attacin E(attE)*	*Antimicrobial peptide*	Hyalophoracecropia	Citrus scab	*C. paradisi* Macf.	[117]
*CitMTSE1*	*Citrus sinensis*	Limonene synthase gene	Black spot	*C. sinensis* (L.) Osbeck	[118]
